# All the Colors of the Rainbow: Diversification of Flower Color and Intraspecific Color Variation in the Genus *Iris*

**DOI:** 10.3389/fpls.2020.569811

**Published:** 2020-10-13

**Authors:** Katarzyna Roguz, M. Kate Gallagher, Esther Senden, Yamit Bar-Lev, Merav Lebel, Roni Heliczer, Yuval Sapir

**Affiliations:** ^1^The Botanical Garden, School of Plant Sciences, Tel Aviv University, Tel Aviv, Israel; ^2^Botanic Garden, Faculty of Biology, University of Warsaw, Warsaw, Poland

**Keywords:** pollination syndrome, flower color evolution, color variation, mating system, pollinator shifts, shelter reward, nectar reward, ancestral trait reconstruction

## Abstract

Floral color plays a key role as visual signaling and is therefore of great importance in shaping plant-pollinator interactions. *Iris* (*Iridaceae*), a genus comprising over 300 species and named after the Greek goddess of the colorful rainbow, is famous for its dazzling palette of flower colors and patterns, which vary considerably both within and among species. Despite the large variation of flower color in *Iris*, little is known about the phylogenetic and ecological contexts of floral color. Here, we seek to resolve the evolution of flower color in the genus *Iris* in a macroevolutionary framework. We used a phylogenetic analysis to reconstruct the ancestral state of flower color and other pollination-related traits (e.g., the presence of nectar and mating system), and also tracked the evolution of color variation. We further explored weather floral trait transitions are better explained by environmental or pollinator-mediated selection. Our study revealed that the most recent common ancestor likely had monomorphic, purple flowers, with a crest and a spot on the fall. The flowers were likely insect-pollinated, nectar-rewarding, and self-compatible. The diversity of floral traits we see in modern irises, likely represents a trade-off between conflicting selection pressures. Whether shifts in these flower traits result from abiotic or biotic selective agents or are maintained by neutral processes without any selection remains an open question. Our analysis serves as a starting point for future work exploring the genetic and physiological mechanisms controlling flower coloration in the most color-diverse genus *Iris*.

## Introduction

Visual floral traits, flower color in particular, are important features that shape plant interactions with the surrounding environment (Schaefer and Ruxton, 2011; Willmer, 2017). Flowers show enormous variation in color among closely related taxa, and even between or within natural populations of the same species (i.e., flower color polymorphism) (Gigord et al., 2001; Warren and Mackenzie, 2001; Narbona et al., 2018; Souto-Vilarós et al., 2018). The factors that drive flower color evolution in highly diverse genera or families, particularly within a species, remain an open area of research.

Flower color is one of the most important characters for signaling to animal pollinators (Schiestl and Johnson, 2013). Therefore, pollinators are often perceived as one of the primary selective agents influencing flower color. Differences in the visual capabilities of pollinators can impose variable selective pressure on flower color, leading to variation (Wester and Lunau, 2017; de Camargo et al., 2019). In some plant species color transitions represent an adaptation to different, sometimes new, suites of pollinators (Armbruster et al., 2000; Fenster et al., 2004). For instance, in some plant genera, color is the best predictor of a transition between insect and bird pollination (Sutherland and Vickery, 1993; Roguz et al., 2018).

While flower color is a central visual cue to animal pollinators, the floral reward also plays a key role in shaping the interaction (Roy et al., 2017; Parachnowitsch et al., 2018; Roguz et al., 2018, 2019). Typically, plants provide a food reward of either pollen or nectar. Of these, nectar is perhaps the most important in an evolutionary sense (Simpson and Neff, 1983; Canto et al., 2011). Although it is a strong attractant, producing nectar is physiologically costly, and thus in several cases, the ability of flowers to produce nectar has been lost (Little, 1983; Dafni, 1984; Sletvold et al., 2016). In some plant families, e.g., Orchidaceae, the presence or absence of a nectar reward may be correlated with flower color. Non-rewarding, sexually deceptive orchids often have brightly colored flowers (Spaethe et al., 2010), while species with nectar often have green or white colored flowers (Duffy and Stout, 2011). The lack of a food reward might result in, or be the result of, the development of new rewarding characters that attract potential pollinators. These new attractants may include changes in flower color and size (Gigord et al., 2001; Vereecken and Schiestl, 2009). For example, night-sheltering reward systems without a food reward are often associated with large, dark flowers (Dafni et al., 1981; Sapir et al., 2005, 2006; Watts et al., 2013; Lavi and Sapir, 2015). Dark petals with their highly absorptive surfaces may result in higher temperatures inside the flower, which may benefit flower visitors (Sapir et al., 2006; Watts et al., 2013). As a result, in such systems flower size and color, but not food rewards, are typically under strong selection (Vereecken et al., 2013; Lavi and Sapir, 2015; Pellegrino et al., 2017).

Flower color changes might also be associated with a shift in plant mating system (Landis et al., 2018). The majority of flowering plants have both male and female reproductive parts, however, the mutualistic interactions with pollinators enable most of these plants to outcross (Goldberg et al., 2010). Shifts in flower color and pollination vectors may result in changes in mating system (i.e., selfing or outcrossing). Some species that rely on pollinators have lost the ability to self-fertilize, becoming self-incompatible, while in other cases the reverse occurred (Landis et al., 2018). Strong selection on floral traits, including color, is expected in self-incompatible taxa relying completely on animal-mediated pollination. Whereas in self-compatible taxa, this animal-mediated selection is expected to weaken since pollinator visitation may no longer be required for reproduction (Anderson and Busch, 2006). Thus, while pollinators often play a critical role in the evolution of flower color, this may not always be the case.

Plants experience a myriad of interactions with animals, both mutualistic and antagonistic. The strength and direction of selection that these agents exert on flower color may differ (Irwin et al., 2003). For example, flower color may have evolved as an adaptation against fungi and/or herbivores (Chalker-Scott, 1999; Chittka and Schürkens, 2001). Irwin et al. (2003) found that herbivores and pollinators exert opposing selection on flower color in *Raphanus sativus*. In this system, because both herbivores and pollinators prefer lighter flowers, dark flowers persist, thus maintaining a stable color polymorphism.

In some taxa, however, there is little or no association between color and interacting animals (Armbruster, 1996). Instead, variation in pigmentation within and among closely related taxa may be maintained by selection related to environmental heterogeneity (Warren and Mackenzie, 2001). The three main groups of pigments responsible for color in plants, flavonoids, carotenoids and betalains, play a functional role in plant physiology (Schaefer and Ruxton, 2011). Flavonoids, for example, are known to function as a response to plant stress caused by drought (Warren and Mackenzie, 2001), cold (Chalker-Scott, 1999; Harborne and Williams, 2000), and nitrogen deficiency (Bonguebartelsman and Phillips, 1995). They also protect plants against damage caused by UV radiation or visible light (Chalker-Scott, 1999; Tanaka et al., 2008). Flower color mediates plant interactions with both the abiotic and biotic environment, and thus exploring the drivers that lead to flower color diversity is of key importance to understand the evolution of plant diversity.

Transitions in flower color are common across the phylogenies of Angiosperm lineages (Streisfeld and Rausher, 2011; Wessinger and Rausher, 2012, 2014; Martins et al., 2016; Roguz et al., 2018). The diversity of flower color among and within these modern lineages suggests that most of these transitions must have been adaptive (Rausher, 2008). While the mutations causing flower color shifts are well understood at the biochemical level (Grotewold, 2006), the broader macro-evolutionary drivers of flower color diversity have only been studied in few plant groups (Landis et al., 2018; Ng and Smith, 2018). To understand these macro-evolutionary forces, we need to explore plant lineages exhibiting a diversity of flower colors.

The genus *Iris* is one of the most diversified genera in Asparagales. *Iris* comprises over 300 species (Royal Botanic and Gardens, 2020), which are widespread throughout the Northern Hemisphere (Goldblatt and Manning, 2008), with the greatest number of endemics occurring in the Mediterranean and Asia (Wilson, 2006). Although some irises are found in mesic or even wetland environments, most species grow in desert, semi-desert, or dry, rocky, montane habitats (Wilson, 2006). Members of this genus display a remarkable variety of flower colors, ranging from extremely dark, purple flowers, through violet and pink, to yellow and white flowers (Mathew, 1989; Sapir and Shmida, 2002; Sapir et al., 2002). This dazzling palette of colors and patterns seen in *Iris* flowers may be associated with their wide variety of life histories, pollination and mating systems, and habitats. Thus irises represent an outstanding model to study evolutionary biology and speciation in plants, especially in the context of flower color (Crespo et al., 2015).

To understand the unusual flower colors and color patterns in irises we investigated the evolution of flower color and several related traits, including pollinator type, nectar reward, and mating system, across the entire phylogeny and geographic range of the genus. By determining the ancestral state of *Iris* traits and comparing it to the traits of modern species, we should be able to shed light on which traits are the causes and which are the effects. To this end, we asked the following specific questions, first, what was the ancestral state of flower color and related traits in *Iris*, and second, are floral trait transitions from ancestral to modern states better explained by environmental or pollinator mediated selection?

## Materials and Methods

### Phylogenetic Tree

To set up our analyses on the evolution of flower color in irises, we first created a phylogenetic tree for the genera, based on a database of sequences. We created the database using six sequences, five plastid genomes (*matK*, *trnL*, *trnK*, *NADPH*, and *rbcL*) and one nuclear internal transcribed spacer (*ITS*). All of the gene sequences were acquired from GenBank (Accession numbers in Supplementary Material 1) and downloaded using the MatPhylobi program (Kranas et al., 2018), which is a command-line tool for constructing taxonomic data sets for phylogenetic inference based on NCBI data. The sequences downloaded represent 227 *Iris* taxa which include 215 species, 10 subspecies and two varieties (see full list and ranks in Supplementary Material 1). Our analysis included all taxa with data available in GenBank. This taxa sampling covers the floral and geographic diversity of the genus. To create the phylogenetic database in MatPhylobi, we seeded *Iris pumila* as the representative *Iris* species and selected *Crocus vernus*, *Morea inclinata*, and *Dietes robinsoniana* as outgroups, based on their previously established sister relationships (Goldblatt et al., 2002; Wilson, 2006). Overall, taxon sampling totalled 429 accessions and total gene coverage was approximately 53.7% (227 *Iris* taxa out of 431), with *matK* having the highest coverage (40.7%) and *ITS* the lowest coverage (9.95%).

To refine the database, all sequences were independently aligned using the multiple alignment program MAFFT (version 7; Kuraku et al., 2013; Katoh et al., 2017) (method = “localpair,” incorporating local pairwise alignment information, maxiterate = 1,000). Subsequently, we imported all of the alignments into Mesquite for visual inspection (version 3.6; Maddison and Maddison, 2018). Poorly aligned positions and divergent regions were eliminated using the Gblocks program (Version 0.91b, Castresana, 2000; Talavera and Castresana, 2007). The trimmed alignments were then concatenated with catfasta2phyml into a single aggregate alignment^1^. For each sequence, we selected the appropriate evolutionary model based on its specific characteristics using ModelTest-NG (version 0.1.3; Darriba et al., 2020; Supplementary Material 2).

Using our refined database of sequences, we used RAxML to generate our final phylogenetic tree. RAxML uses a series of maximum-likelihood (ML) tests to generate the tree (version 8.0; Stamatakis, 2014). To find the best phylogenetic tree, we used a bootstrap analysis with 1,000 replicates. Only bootstrap values (i.e., the probability that respective groups of taxa are present in the true phylogeny) higher than 50% were presented on the tree.

### Floral and Habitat Characters

Once we had our phylogenetic tree, we prepared a database describing the diversity of irises, including 16 traits related to flower color, reproduction, habitat and distribution (Table 1). A majority of these traits were determined using Mathew (1989), which is a compilation of information about irises around the world. Additional sources included regional floras, the scientific literature, as well as internet sources^2^ ^3^. Table with all data available in Supplementary Table 1.

**TABLE 1:** Results of phylogenetic signal analysis.

Trait	Trait type	Phylogenetic signal	
Bi-colored flowers	Categorical binnary	Estimated D:0.52	Probability of E(D_r_):0 Probability of E(D_B_):0.001
Color polymorphism	Categorical binnary	Estimated D:0.70	Probability of E(D_r_):0.002 Probability of E(D_B_):0
Continuous flower color variation	Categorical binnary	Estimated D:0.89	Probability of E(D_r_):0.114 Probability of E(D_B_): 0
Beard on the falls	Categorical binnary	Estimated D:-0.36	Probability of E(D_r_):0 Probability of E(D_B_):0.99
Crest on the falls	Categorical binnary	Estimated D:-0.30	Probability of E(D_r_):0 Probability of E(D_B_):0.95
Color spot on the falls	Categorical binnary	Estimated D:0.75	Probability of E(D_r_):0 Probability of E(D_B_):0
Diameter	Continuous	Phylogenetic signal K : 0.0022662 Phylogenetic signal lambda : 0.616142	*P*-value: 0.159 *P*-value: 0.000
Mating system	Categorical binnary	Estimated D:-0.14	Probability of E(D_r_):0 Probability of E(D_B_):0.001
Nectar production	Categorical binnary	Estimated D:	Probability of E(D_r_):0 Probability of E(D_B_):0.001
White morphs	Categorical binnary	Estimated D:0.66	Probability of E(D_r_):0 Probability of E(D_B_):0.95

**For all of the binary traits (all but diameter) the signal is expressed as a D-value (probability of E(D_r_): a p value, giving the result of testing whether D is significantly different from one, probability of E(D_B_): a p value, giving the result of testing whether D is significantly different from zero; caper package, “phylo.d” function). For the continuous trait (diameter) the strength of the phylogenetic signal is expressed as the Blomgergs’s K and Pagels λ (phytools package in R, version 0.5–20; Revell, 2012).**

**FIGURE 1:**
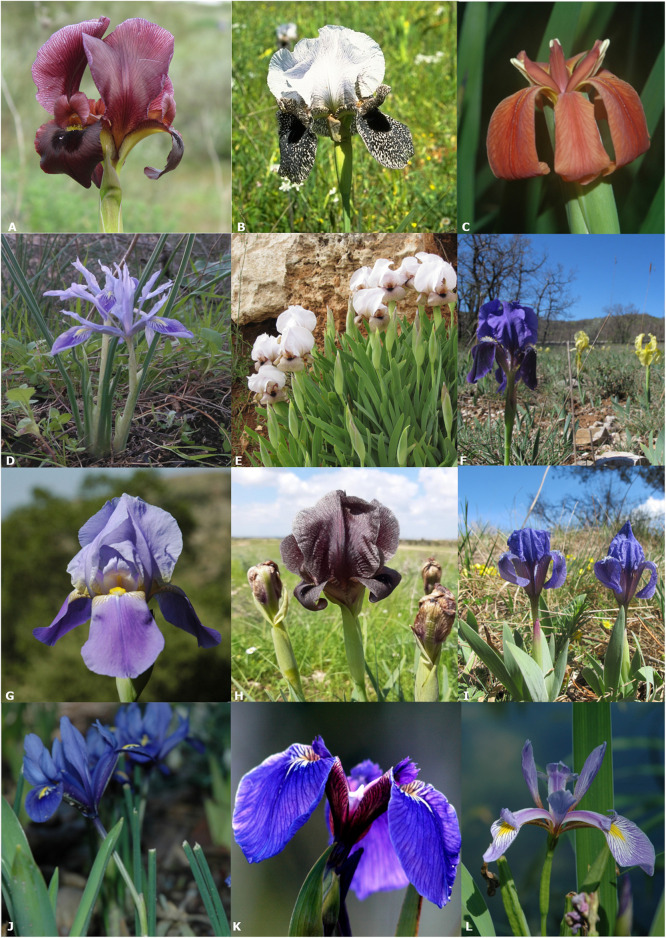
Flowers of selected *Iris* species [**(A)** *I. atropurpurea*, **(B)** *I. bismarckiana*, **(C)** *I. fulva*, **(D)** *I. historio*, **(E)** *I. loretti*, **(F)** *I. lutescens*, **(G)** *I. mesopotamica*, **(H)** *I. petrana*, **(I)** *I. pumila*, **(J)** *I. reticulata*, **(K)** *I. setosa* **(L)** *I. virginica*].

*Iris* flowers have six sepals, which are usually divided into two types: three falls that droop downwards and three standards that are upright. These two sepal-types often have distinct characteristics (Figure 1).

We assessed flower color and eight color-related traits. Because of the wide range of taxa and variation in literature sources regarding those taxa, our first color metric was a simple assessment of flower tepals color based on human perception. Although UV reflecting flower parts may play an important role in communication with pollinators, we were not able to include this information in our study due to a lack of relevant data. The availability of flower reflectance data of any sort is limited among *Iris* taxa and do not allow genus-level analysis. For each taxon, the color was assessed and categorized into seven flower color categories: maroon, orange, pink, purple, red, yellow and white. In irises the differences between blue, violet and purple flowers are vague, therefore we coded species with flowers in these colors as purple. Polymorphic taxa fell into several categories (e.g., *I. lutescens* with blue and yellow morphs). Taxa with bi-colored flowers (e.g., *I. narbuttii* with yellow standards and violet falls) were coded as representing two categories. Second, we assessed flower pigment. Taxa were categorized as having anthocyanins (pink, purple, or red flowers) or carotenoids (orange and yellow flowers) as the major pigment, or lacking anthocyanins flavonoids and carotenoids (white or creamy specimens). As in the previous flower color trait, polymorphic and bi-colored flowers were coded into multiple categories. Several previous studies have used these pigment categories, which allow comparisons among flower taxa while eliminating differences related to color perception or habitat influence (Arista et al., 2013; Smith, 2015; Ellis and Field, 2016; Landis et al., 2018).

Flower color polymorphic species with white-flowered morphs (i.e., lacking anthocyanins and carotenoids) were assigned based on the colored morph (with pigment) to a pigment trait category as has been done in previous studies (Smith, 2015; Ellis and Field, 2016; Landis et al., 2018). Therefore, to capture the presence of white morphs, we added a white/non-white binary trait category. We also had binary trait categories for bi-colored/not bi-colored flowers, poly/monomorphic flowers (not including white morphs), and continuous/non-continuous flower colors (e.g., where color morphs occur across a continuous color gradient instead of discrete color morphs, as is the case in *I. petrana*). All species with continuous flower colors were also described as having polymorphic flowers. Finally, we also assessed the presence or absence of three traits that contribute to the overall visual display of *Iris* flowers: beard, crest, and spot. In the centre of the fall, irises either have a hairy or bristly tuft called a beard, or a cockscomb-like crest. Additionally, many *Iris* taxa have a signal patch (*hereafter* spot) of a different or stronger color on the fall (Mathew, 1989).

We also assessed several traits related to flower attraction and reproduction that potentially played a role in the evolution of *Iris* flowers: corolla diameter, pollinator type, mating system (self-compatibility vs. self-incompatibility), and presence of nectar. Apart from diameter, the rest of these data were ascertained either from the published literature or from personal communication (Supplementary Table 1). In most cases, pollinator type was not described to taxon level, therefore to be conservative we included only two broad groups, insects or birds.

Finally, for all studied taxa with available information, we assessed habitat type, elevation (maximum height), and geographical range. There were 10 habitat categories, with some taxa falling into several categories (e.g., desert and stony slope). Irises are found across the entire Northern Hemisphere and we identified the specific regions in which each taxon can be found. To assess whether there is any pattern relating flower color to geographical range, we used the geographic data to overlay the proportion of taxa with different flower color categories onto a QGIS map using QGIS 3.10.5 (Szczepanek, 2012; QGIS Development Team, 2020). For this visualization, each pie chart is located in the center of the specific geographical region, as calculated by QGIS.

Overall, we have flower color data for all 227 taxa. Coverage of other traits is less complete: corolla diameter 92%, pollinator type 56%, mating system 51%, and nectar availability 59%. We were able to capture data about habitat type and geographical range for all taxa, but only found elevation data for 90% of taxa.

It is important to mention that our study is biased by the sources and quality of the data we were able to obtain to populate our trait table. Ideally, we would have liked to have more resources and resolution around some of these traits, particularly around the reproductive systems of the studied irises. Also, although flower scent and color may be derived from the same biosynthesis pathways (Delle-Vedove et al., 2017) and scent plays an important role in pollinators attraction (Dormont et al., 2019) we did not include scent in our analysis due to a general lack of data regarding iris floral volatiles. That said, we are confident that we captured what data is available for these taxa and have made our inferences on that basis.

### Ancestral State Reconstruction and Phylogenetic Signal

We used our phylogenetic tree and trait databases to determine the ancestral state of flower color and related traits in *Iris.* The ancestral states were inferred from ultrametric tree, generated using the *chronos* function in “ape” package (with the age of the tree set to one, value of smoothing parameter lambda = 0 based on log-likelihoods; Paradis et al., 2004). For each trait, we first determined the appropriate transition probability model, choosing among ER—equal rate, SYM—symmetrical rate, and ARD—all-rates different, using a log likelihood ratio analysis. In all cases, the ARD transition probability model was chosen because it had the highest likelihood value. However, in our analyses testing the ancestral states of bi-color, continuous, and polymorphic flowers our results suggested that the ARD model was overfitted (number of detected trait changes with the ARD model was several million vs. a thousand in the ER model), therefore for these analyses we used the ER model instead. To compute the total number of character changes between all states of binary trait categories, we used the make.simmap function in the phytools package (100 simulations across the tree, Revell, 2012).

For polymorphic traits (i.e., flower color and pigment), we inferred the ancestral character states with the R package *corHMM* (Beaulieu et al., 2013) using the “rayDISC” function, which specifically accommodates polymorphic characters. For all of the binary trait categories, we estimated the ancestral character states using a continuous-time Markov chain model (Mk model, phytools package). For the ancestral state reconstruction of the continuous trait, diameter, we used the FastAnc function in the phytools package (version 0.5–20; Revell, 2012).

Testing the strength of the phylogenetic signal reveals a tendency for related taxa to resemble each other more than taxa drawn at random from the same tree. For all of the binary trait we measured a D-value (Fritz and Purvis, 2010), which is a measure of phylogenetic signal dedicated to this kind of dataset (caper package, “phylo.d” function, Orme et al., 2013). The strength of the phylogenetic signal on continuous data was calculated as the Blomgergs’s K (Blomberg et al., 2003) and Pagels λ (Pagel, 1999) (phytools package in R, version 0.5–20; Revell, 2012).

## Results

### Phylogenetic Tree

Most of the described large-scale phylogenetic relationships found in previous studies were recaptured in our final tree (Wilson, 2004, 2009; Wilson et al., 2016; Jiang et al., 2018). That said, we decided to exclude *Iris darwasica* from the analysis, because of conflicting nesting results between our study and others. Specifically, we found that *I. darwasica* was assigned to the subgenus *Limniris*, but according to previous studies and floral characters this taxon belongs to the subgenus *Iris*, section *Regelia* (Khassanov and Rakhimova, 2012). Leaving such a wrongly assigned taxon in our tree may have biased all subsequent analyses.

There were some differences in the topology observed between the six sequence-specific trees and the final summarizing tree. For example, in two of the six trees that were rooted on *Crocus vernus* (*matK* and *ITS*), the outgroup *Dietes robinsoniana* resolved as nested within the *Iris* subgenus *Limniris* (low bootstrap; *hereafter* Bp).

**FIGURE 2:**
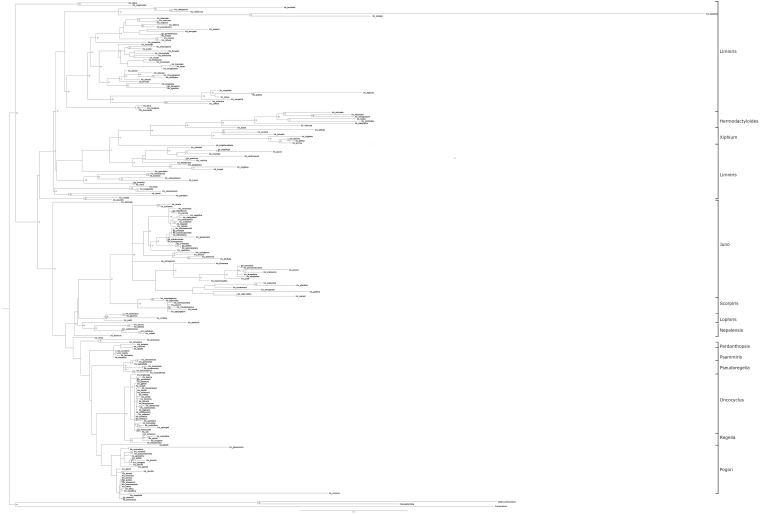
Maximum likelihood tree inferred from analysis of combined five plastid genomes (*matK*, *trnL*, *trnK*, *NADPH* and *rbcL*) and one nuclear internal transcribed spacer (*ITS*) sequences. The bootstrap values are given along the branches (only values > 50 presented).

As in previous studies, the topology of our final phylogenetic tree has two major subgenera (*Limniris* and *Iris*) and five minor subgenera (*Hermodactyloides, Nepalensis, Pardanthopsis, Scorpiris*, and *Xiphium*). All but *Limniris* were resolved as monophyletic. Within *Limniris*, there were two sections, *Limniris* (71 Bp) and *Lophiris*, with the first section containing the majority of the taxa belonging to the genus. Within the subgenus *Iris*, there were six sections: *Pardanthopsis* (99 Bp), *Psammiris* (56 Bp), *Pseudoregelia* (99 Bp), *Oncocyclus* (86 Bp), *Regelia* (87 Bp), *Hexapogon* (86 Bp), and *Pogon* (Figure 2).

A full discussion of the final tree and contributing sub-trees is presented in Supplementary Material 3.

### Floral and Habitat Characters

**FIGURE 3:**
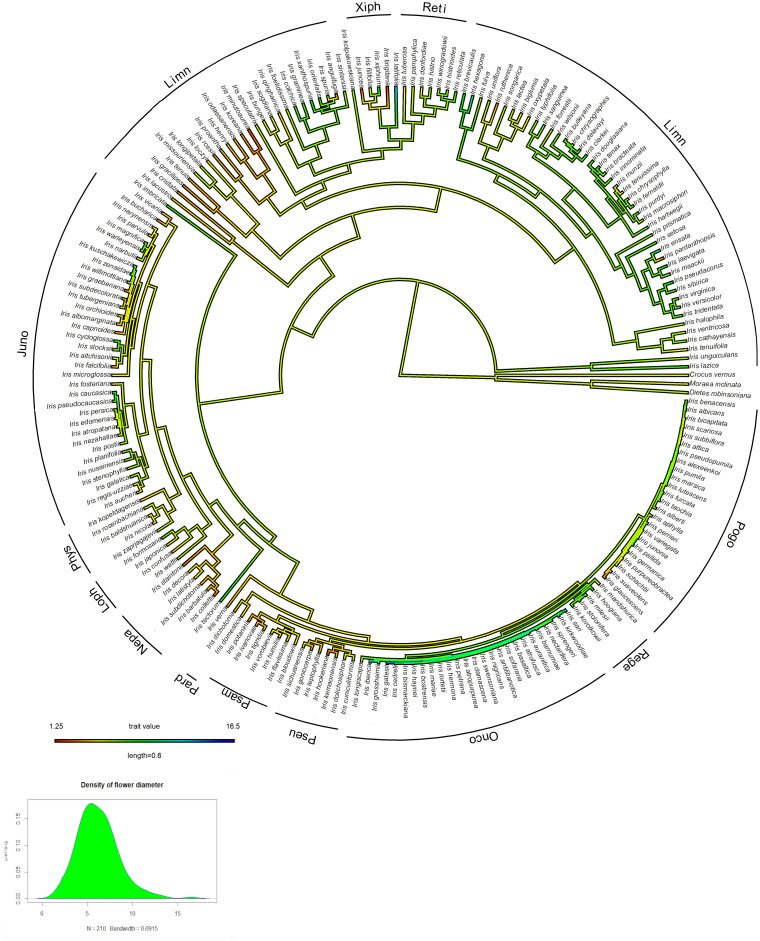
Maximum-likelihood ancestral state reconstruction of corolla diameter [A] and histogram showing representation of the distribution of the variables [B].

Of the 226 *Iris* taxa included in this analysis, 49.3% had flowers in shades of purple and 24.5% had yellow flowers. The rest were distributed among maroon (14%), white (11%), pink (4%), orange (0.5%), red (0.5%). Most of the studied taxa were categorized as having anthocyanins (80%) and a third were characterized as having carotenoids (33%), with several taxa containing both pigments. Approximately 43% of the genus has white flower morphs or flower-parts (i.e., white standards or falls). There were 35 taxa categorized as having bi-colored flowers, 35 taxa categorized as having polymorphic flowers, and 23 taxa with continuous flower color variation. More than half of the *Iris* taxa have a crest (141 taxa), while over a third (85 taxa) have a beard; also, more than half of *Iris* taxa have a spot (143 taxa). *Iris* flowers range in diameter between 1.25 and 16.5 cm (MEAN ± SD: 6.2 ± 2.3 cm) (Figure 3 and Supplementary Table 1).

The majority of *Iris* taxa are pollinated by insects, primarily bees (members of *Andrena, Anthophora, Apis, Colletes, Emphoropsis, Eucera, Hylaeus, Lasioglossum, Nombus, Osmia, Tetralonia*, and *Xylocopa*). Several *Iris* taxa, however, are also pollinated by flies (e.g., *I. bracteata, I. gracilipes, I. palaestina*) and butterflies (e.g., *I. fulva*). Hummingbird pollination was observed in four *Iris* taxa: *I. cristata, I. fulva, I. hexagona*, and *I. missouriensis*. Most *Iris* taxa produce nectar and are self-compatible. The majority of taxa that do not produce nectar are also self-incompatible, have a beard, and belong to the *Oncocyclus* and *Regalia* sections.

Taxa with purple and yellow flowers are almost equally distributed on all continents, although yellow morphs are rare in South Asia. Polymorphic and bi-colored taxa are more prevalent in the Middle East than in other regions, and bi-colored purple-white species are completely absent from North America (Supplementary Figure 1).

### Ancestral States of Floral Traits, Their Transitions, and Phylogenetic Signal

**FIGURE 4:**
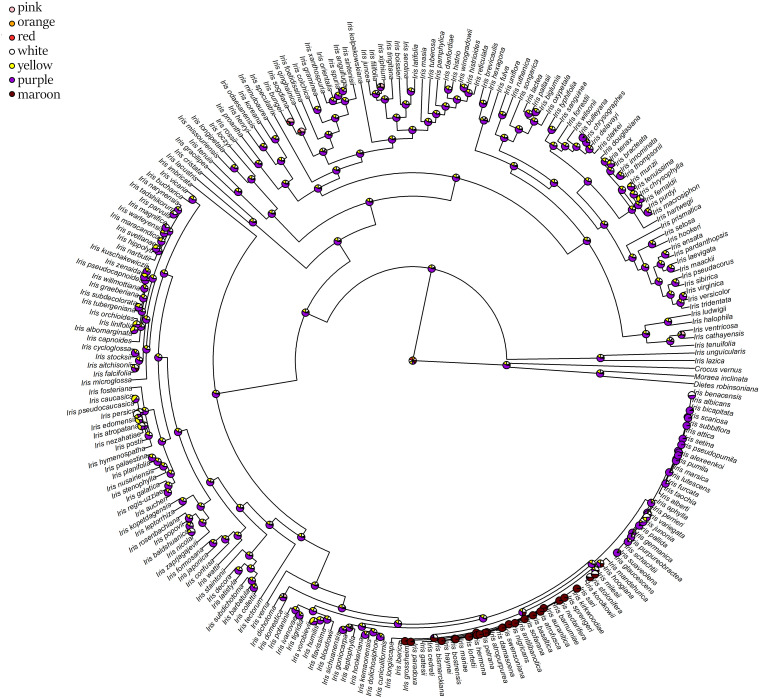
Estimation of ancestral states of flower colors among studied *Iris* taxa calculated using maximum likelihood across the posterior distribution.

**FIGURE 5:**
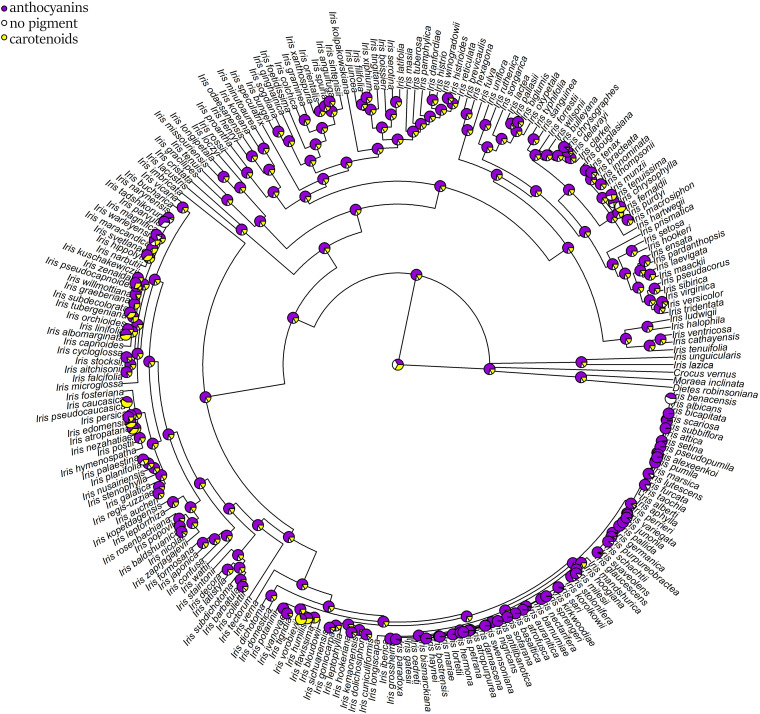
Estimation of ancestral states of flower pigments among studied *Iris* taxa calculated using maximum likelihood across the posterior distribution. White and creamy flowers were coded as lacking anthocyanins flavonoids and carotenoids and are represented as white; maroon, purple, pink, or red flowers were coded as having anthocyanins and are represented in purple; yellow or orange flowers were coded as having carotenoids and are represented in yellow.

**FIGURE 6:**
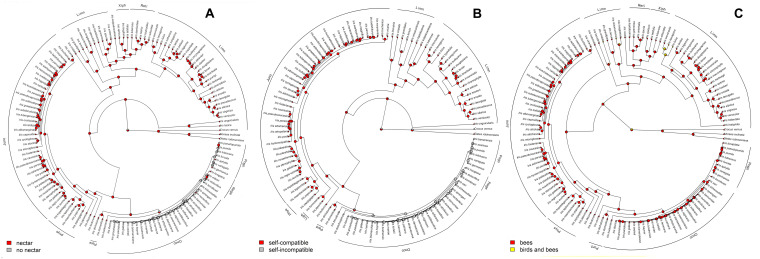
Summarized stochastic mapping of mating system (self-compatibility vs. self-incompatibility), **(A)** presence or absence of nectar **(B)**, and pollinator type (insect vs. insect and bird) **(C)** in the genus *Iris* prepared using All Rates Different model with 1,000 iterations. Pie charts represent the proportion of the iterations showing either presence or absence of nectar at any given node.

The ancestral flower color of *Iris* was most probably purple (Figure 4) and anthocyanin-based (Figure 5), without the ability to produce white flower morphs (Supplementary Figure 2). Most internal nodes also exhibited purple flowers with anthocyanin pigments (Figures 4, 5). Having color-monomorphic flowers, where all parts of the flower are the same color, was likely the ancestral state among *Iris* and remained so in the early nodes. Taxa with polymorphic, continuous, or bi-colored flowers all seem to be derived states that have arisen and been lost several times (Supplementary Figure 3). The most recent common ancestor of *Iris* likely had flowers with a crest and a spot (Supplementary Figure 4), and was self-compatible, insect-pollinated, and nectar-rewarding (Table 2A and Figure 6).

**TABLE 2A:** Results of simulated stochastic character mapping on *Iris* phylogenetic tree (all-rates different selected as transition probability model).

	Beard/No beard	Bi-colored (1) /no bi-colored (0)	Continuous color (1) /no continuous color (0)	Crest (1) /no crest (0)	Insect (1) /insect and bird (0)	Monomorphic (1) /polymorphic (0)	Nectar (1) /no nectar (0)	Self-compatible (1) /self-incompatible (0)	Spot (1) /no spot(0)	White forms (1) /no white forms (2)
Average number of changes between states Number of specific transitions	9.72	56.2	30.6	10.4	11.1	64.0	19.9	2.4	1,582	1,098
1 −> 0	5.05	24.3	8.54	6.26	5.17	38.5	6.32.	2.07	780	548
0 < − 1	4.67	32.0	22.1	4.16	5.96	25.5	13.6	0.33	802	550
Proportion of time spent in the state 1	0.09	0.13	0.08	0.83	0.91	0.83	0.97	0.95	0.51	0.56
Proportion of time spent in in the state 0	0.91	0.87	0.92	0.17	0.09	0.17	0.03	0.05	0.49	0.44

**Number and time of transitions between studied Iris characters identified with the use of make.simmap function in the phytools R package. The estimations are based on 100 trees with a mapped character.**

Flower color and pigment are variable, with most of the color and pigment transitions being from yellow/carotenoid-dominated flowers to purple/anthocyanin-dominated flowers. The transitions between purple and white were also common. Of the taxa involved in these purple-white shifts, a majority represent bi-colored flowers, where either the fall or standard is white (Table 2B). The ability to produce white flower morphs is widespread across the phylogenetic tree (1,098 transitions on average; *hereafter*—average value based on 100 simulations), but labile, with several changes back and forth. In contrast, having monomorphic flowers with a crest persisted for most of the time. The small number of transitions between color monomorphic or polymorphic, and between having a crest or a beard suggests that these traits tend to be conserved. The proportion of time that *Iris* lineages have had a beard is comparatively short and this trait probably evolved once in a common ancestor of the subgenera *Pogon, Oncocyclus, Pseudoregelia, Regelia, Psammiris*, and *Pardanthopsis*, and two times more recently for *I. falcifolia* and *I. imbricate*. Unlike the crest and the beard, the presence of a spot varied more across taxa, with several changes among closely related taxa (Table 2A).

**TABLE 2B:** Matrix containing the maximum likelihood estimates of the transition rate of color and pigment on *Iris* phylogenetic tree (all-rates different selected as transition probability model).

	Pink	Orange	Red	White	Yellow	Purple	Maroon
Pink	NA	15	0.76	0.76	0.76	30.5	0.76
Orange	64.6	NA	23.2	0.76	0.76	0.77	0.76
Red	16.3	0.76	NA	0.76	0.76	0.76	0.76
White	0.76	0.76	0.76	NA	0.76	0.76	10.2
Yellow	0.76	0.76	0.76	0.76	NA	100	0.76
Purple	1.81	0.76	0.76	22.6	9.95	NA	0.76
Maroon	27.8	0.76	0.76	0.76	39.7	27.9	NA

		**Ant**		**No & and car**			**Car**

Ant		NA		13.9			22.9
No ant & car		100		NA			0.76
Car		100		0.76			NA

**The maximum likelihood values identified with the use of rayDisc function in the corHMM R package. The estimations are based on 1,000 trees (pigment abbreviations: ant, anthocyanin-based; no & and car lacking anthocyanins flavonoids and carotenoids; car, carotenoids-based.**

The ability to self-pollinate and produce nectar was conserved for most of the time (Table 2A), and only lost once in the common ancestor of the subgenera *Pogon* and *Oncocyclus*. Both self-compatibility and the ability to produce nectar were regained several times. Even though an overwhelming majority of taxa are, and always have been, insect-pollinated, we found two independent shifts toward bird pollination (Figure 6), as well as several reversals from birds to insects (Table 2A).

Across the *Iris* lineage, we found all the studied binary traits to be phylogenetically conserved. Among them, the distribution of the states for the half of the traits (presence of bi-colored flowers, color polymorphism, continuous flower color and color spot on the falls) proofed to be distributed as expected under the Brownian motion model of evolution (0<D < 1). For the second half (presence of beard, crest, nectar, or mating system) the distribution of the states was more phylogenetically conserved than the Brownian expectation (D < 0). We also found a relatively strong phylogenetic signal for the flower diameter, however the significant value was obtained only for Pagel’s λ (Table 1).

## Discussion

*Iris* flowers display a wide diversity of colors and color patterns among and within species. The diversity seen in modern irises is the result of several changes over evolutionary history in the genus. In this study, we reconstructed the traits of the most recent common ancestor of the *Iris* genus. This ancestor likely had monomorphic, one-colored purple flowers, with a crest and a spot on the falls. The flowers were likely insect-pollinated, nectar-rewarding, and self-compatible. Since then, the genus has diverged to include over 300 taxa that exhibit a wide range of colors and patterns, including polymorphic, continuous, and bi-colored flowers. Additionally, some taxa now have a beard, instead of a crest, have lost the spot (and sometimes regained it), and some are now self-incompatible and rewardless. There have been a few shifts from insect-pollination to bird-pollination, and occasionally back again. Thus, by comparing these derived states to the ancestral state, and placing them in the context of the *Iris* distribution and reproductive system, we can infer some of the drivers of floral color diversity in *Iris*.

Flower color is the most variable trait of those we studied in the genus *Iris*. Some of that diversity is likely the result of mutations, which can cause up- or down-regulation of specific genes, leading to differences in the amount of synthesized pigment and in color shades (Durbin et al., 2003). The correlation between flower color diversity and anthocyanin synthesis is common not just in *Iris*, but across many genera, e.g., *Petunia* and *Ipomea* (Durbin et al., 2003). Unlike some of the other groups, however, the flower color diversity in *Iris* is not just related to the presence or absence of anthocyanin pigments, but also the result of variation in colors produced by anthocyanins (Warren and Mackenzie, 2001). Moreover, in *Iris* we also observed many cases of bi-colored flowers, which may be the result of tissue-specific variation in anthocyanin production, or the loss of anthocyanins in the standard or the fall.

Color polymorphism, continuous coloration, and bi-colored flowers arose multiple times across the *Iris* phylogeny, and many are maintained to this day (Wang et al., 2016). The maintenance of color polymorphism is not common in other genera. Typically, one of the color morphs will eventually be lost and the taxa will again be monomorphic (Ellis and Field, 2016). The maintenance of stable color polymorphism, and other color variations, in *Iris* may be caused by an absence of selective disadvantage on color in these taxa or by stabilizing selection exerted by multiple agents (reviewed in Sapir et al., in review). For example most populations of *I. lutescens* and *I. pumila* are polymorphic and neutrally distributed in space (Wang et al., 2016), but in some populations color variation may be due to divergence (Souto-Vilarós et al., 2018). Several factors may maintain this neutral distribution, including environmental heterogeneity (Tucić et al., 1998). Additionally, while selection should lead to the fixation of one color morph, polymorphism may also be maintained over the long-term due to perennial life history and vegetative reproduction, which cause generation overlap in many *Iris* species (Gray and McKinnon, 2007).

Almost half of the studied *Iris* taxa produce white morphs, which is a common color-transition in many angiosperm taxa, resulting from shut-down of genes in the pigment biosynthesis pathway (Wessinger and Rausher, 2012). However, the modern *Iris* taxa with (only) white-colored flowers can be found in the subgenera *Scorpiris* and *Limniris*. For some *Iris* species white flowers may be an evolutionary dead-end, without possibility for reversal. Pigment loss may alter pollinator attractiveness (Waser and Price, 1981) or reduce the capacity to deal with environmental stress, such as drought (Ellis and Field, 2016), or a combination of the two (Warren and Mackenzie, 2001).

Like the high diversity of flower color and patterns seen across *Iris* taxa, corolla diameter seems to be fairly variable across the phylogeny. The visual attractiveness of flowers is substantially related to flower color and size. Although large flowers are costly (Teixido et al., 2016; Roddy, 2019), some of the species with large flowers in the genus *Iris* grow in desert or semi-arid habitats. This suggests that pollinator-mediated selection is more important in driving the evolution of large flowers (Lavi and Sapir, 2015; Souto-Vilarós et al., 2018). The strategy to produce big, showy, self-compatible flowers with lots of nectar is found in most *Iris* taxa and may increase mating success, especially when florivory is not a threat (Ghara et al., 2017). While positive pollinator-mediated directional selection on flower size may be a factor in some *Iris* taxa (Lavi and Sapir, 2015; Souto-Vilarós et al., 2018), this is not always the case (Ishii, 2005; Pellegrino, 2015).

Most *Iris* species are pollinated by insects, but in a few cases, shifts to bird pollination evolved. Several taxa of *Iris* have blue-violet or purple flowers, which tend to be associated with bee visitation because bees tend to have an innate preference for the blue range of wavelengths (Lunau and Maier, 1995; Dyer et al., 2015). Bees also have an innate preference for yellow flowers, and thus the common shift from purple to yellow flowers in *Iris* may be maintained by bee preferences (Giurfa et al., 1995; Lunau and Maier, 1995; Spaethe et al., 2001). This innate preference for both purple and yellow flowers may explain the equal seed set between color morphs in some polymorphic *Iris* populations (Imbert et al., 2014a, b; Souto-Vilarós et al., 2018).

Bird-pollinated flowers tend to be red or orange (Rodríguez-Gironés and Santamaría, 2004; Cronk and Ojeda, 2008), and these two colors have arisen several times in the *Iris* phylogeny. Red coloration may be a good predictor of bird-pollination in irises, but not vice-versa. There is only one taxon with red flowers, and it is bird-pollinated (*I. fulva*, Emms and Arnold, 2000; Martin et al., 2008), but other bird-pollinated species are not red (e.g., *I. missouriensis*, Lyon, 1973). This suggests that color is likely not the major trait driving pollinator-shifts from insects to birds in *Iris*. All the bird-pollinated taxa have wide, open and flat flowers that produce dilute nectar (Wesselingh and Arnold, 2000). Being able to access the flower (wide open, flat flowers) and obtain the preferred food reward (diluted nectar) seems likely to be the trigger for the transition to bird pollination. Shifting from purple to red flowers may be relatively easy because the anthocyanin biosynthesis pathway is relatively flexible (Warren and Mackenzie, 2001; Albert et al., 2011). Bird-driven transitions from purple to red flowers, however, would likely be countered by insect pollination because insects often prefer blue-violet (Lunau and Maier, 1995; Dyer et al., 2015) and yellow flowers (Giurfa et al., 1995; Lunau and Maier, 1995; Spaethe et al., 2001). This may explain why two out of the three extant bird-pollinated flowers are purple, and not red.

We found a strong signal for the association among floral structure, reward, and mating system. All taxa have either a crest or a beard, but never both. Although the ancestral *Iris* probably had a crest, the beard replaced the crest relatively early in the common ancestor of *Oncocyclus, Pogon*, and *Regalia*. Around the same time, this group of subgenera also lost the ability to produce nectar and became self-incompatible. Once the transition to a beard and self-incompatibility occurred in this section, it never changed back. In contrast, nectar production was regained several times. We also found, that white-purple and purple flower color, which are commonly found among members of these subgenera, are associated with self-incapability and a lack of nectar. While the association of these traits and their strong phylogenetic signal suggests the evolution of a monophyletic pollination syndrome, it is important to note the sampling of these traits was not similar across all species.

Nectar production requires regular and consistent water availability. In the dry regions inhabited by *Oncocyclus, Pogon*, and *Regalia* irises, water limitation could have been the agent of selection against nectar production, leading to the loss of this trait in their common ancestor. Once these irises became nectarless, the question becomes: how did they attract insect pollinators? Potentially, this is when the night-sheltering reward system, that is well-described in the *Oncocyclus* group, arose (Sapir et al., 2005; Monty et al., 2006; Vereecken et al., 2013; Watts et al., 2013). *Oncocyclus* irises are primarily pollinated by male *Eucera* bees (*Apidae, Eucerini*) that shelter in the flowers overnight (Sapir et al., 2005, 2006; Watts et al., 2013). Pollination occurs as the male bees visit multiple flowers before choosing the flower in which they will spend the night (Sapir et al., 2005; Monty et al., 2006). While it is possible that bees slept in the flowers before nectar was lost, after nectar-loss the sheltering component became the only reward. Thus, shelter may have become a replacement mechanism to attract pollinators.

The loss of nectar is associated with the transition from crest to beard. If a crest serves as a nectar guide, there will be no selection to maintain it in nectarless species. Instead, a beard might have selected by its advantage in alternative reward types, such as the derived sheltering-reward system. The role of the beard remains debatable, but it would be interesting to test whether the presence of a beard changes the airflow over the fall and impacts the rate of warming in these flowers, or whether it contributes for pollen deposition on stigma.

Another open question is why self-compatibility only arose in the closely related *Oncocyclus, Pogon*, and *Regalia* (except for *I. tenuis).* The loss of self-compatibility seems risky, but perhaps it was necessary to maintain genetic diversity. Most of the taxa in these subgenera occur in highly variable habitats in the Middle East and Western Asia. In a complex habitat mosaic, with patchy and extreme environmental conditions, being self-compatible may be maladaptive. Self-compatibility would naturally lead to selfing and inbreeding, thus reduce genetic diversity. Maintaining genetic diversity is particularly important for plants in highly variable and stressful conditions. It facilitates quick adaptation and increased population persistence, as a sort of insurance. Indeed, most of these taxa have highly variable flower traits, most notably in terms of color.

The nectarless *Oncocyclus, Pogon*, and *Regalia*, tend to have high levels of color polymorphism, continuous color variation, and bi-colored flowers. Within-species variation in flower color may be important in deceptive systems, since it impedes the learning ability of pollinators and leads to negative frequency-dependent selection (Smithson and MacNair, 1997; Gigord et al., 2001; Imbert et al., 2014a). Another strategy to maintain the attractiveness of no-food rewarding species is flowering early in the season and attracting naïve pollinators (Imbert et al., 2014a). In any case, it is surprising that more taxa in these subgenera did not regain nectar production. Comparative studies have shown that nectarless taxa in general (Aragon and Ackerman, 2004; Sletvold et al., 2016), and *Iris* taxa specifically (Lavi and Sapir, 2015; Souto-Vilarós et al., 2018), are much more pollen limited than nectar producing taxa.

Irises cover a broad geographic range across a mosaic of habitat types, environmental stresses and pollinator types, which is likely the ultimate cause of the observed flower diversity. In general, some *Iris* taxa seem to have strong pollinator-mediated selection on floral traits, such as color and size. In other taxa, however, the environment seems to play a stronger selective role in one or more of these traits. The diversity of flower colors we see in *Iris*, likely represents a trade-off between conflicting selection pressures (Souto-Vilarós et al., 2018). Whether changes in flower color are the result of neutral processes without any selection, or whether these changes are tightly maintained by abiotic or biotic selective agents, remains an open question.

## Data Availability Statement

All datasets generated for this study are included in the article/Supplementary Material.

## Author Contributions

YS and KR conceived and designed the research. KR and MG performed analyses. KR, MG, and YS wrote the manuscript, with contributions from all authors. All authors collected the data.

## Conflict of Interest

The authors declare that the research was conducted in the absence of any commercial or financial relationships that could be construed as a potential conflict of interest.

## Footnotes

1 https://github.com/nylander/catfasta2phyml

2 http://www.signa.org

3 http://www.wiki.irises.org

## Dedication

This article is dedicated to Brian Mathew, leader in Iris taxonomy and ornamental bulb plants.

## Supplementary Material

Supplementary Figure 1 Map showing the total proportion of pigments among studied *Iris* taxa and the distribution of color morphs across the geographical range.

Supplementary Figure 2 Summarized stochastic mapping of the presence of the white/no-white flowers in the genus *Iris* using All Rates Different model with 1,000 iterations. Pie charts represent the proportion of the iterations showing either presence or absence of the white/no-white flowers at any given node.

Supplementary Figure 3 Summarized stochastic mapping of the presence of the poly/monomorphic **(A)**, continuous/non-continuous color **(B)**, bi-colored/not bi-colored **(C)** flowers in the genus *Iris* using All Rates Different model with 1,000 iterations. Pie charts represent the proportion of the iterations showing either presence or absence of the studied traits in flowers at any given node.

Supplementary Figure 4 Summarized stochastic mapping of the crest **(A)**, beard **(B)**, or spot **(C)** presence/absence in the genus *Iris* using All Rates Different model with 1,000 iterations. Pie charts represent the proportion of the iterations showing either presence or absence of studied trait at any given node.

Supplementary Table 1 Data table. Data include color and floral traits, habitat and source of data.

Supplementary Material 1 Accession numbers of the gene sequences acquired from GenBank.

Supplementary Material 2 Evolutionary models selected with the use of ModelTest-NG program.

Supplementary Material 3 The contributing sub-trees of *Iris* genus; results and discussion of the phylogeny.
