# Supplementary figures and images for: All the Colors of the Rainbow: Diversification of Flower Color and Intraspecific Color Variation in the Genus Iris
Source: Front Plant Sci. 2020 Oct 13;11:569811. doi: 10.3389/fpls.2020.569811 (PMC7588356; doi:10.3389/fpls.2020.569811)

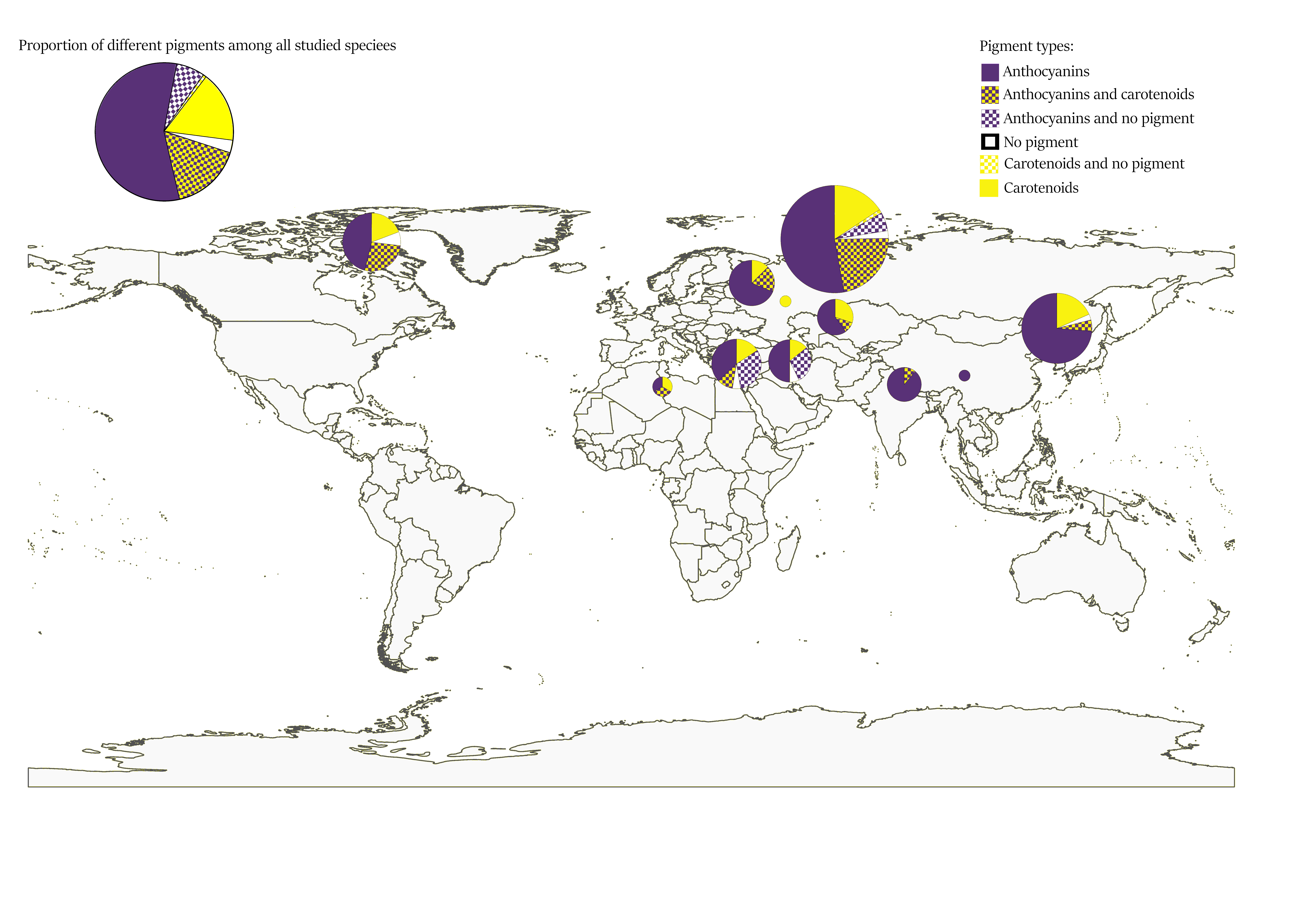

Supplement: Supplementary Figure 1 — Map showing the total proportion of pigments among studied Iris taxa and the distribution of color morphs across the geographical range. [file Image_1.JPEG]

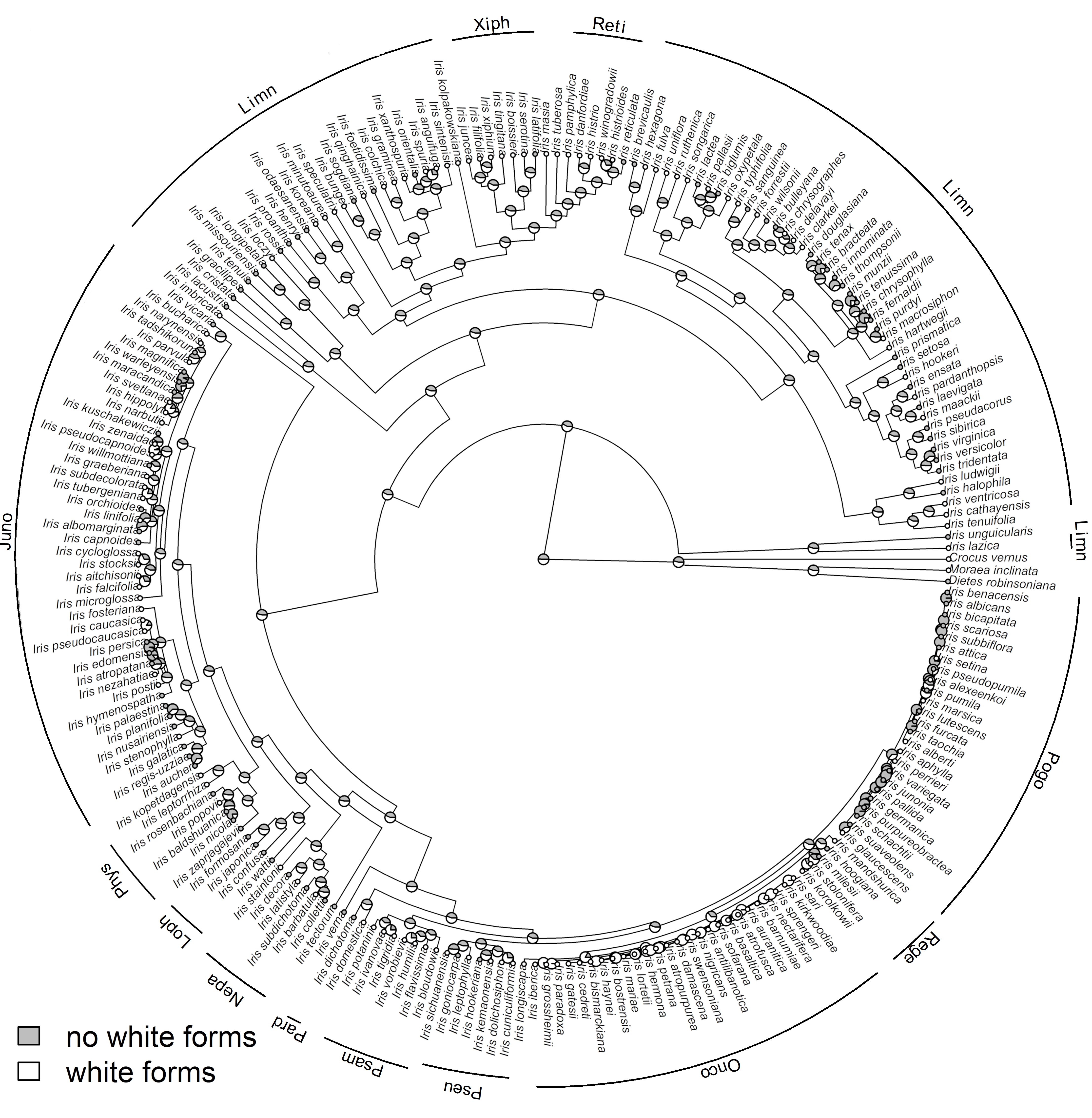

Supplement: Supplementary Figure 2 — Summarized stochastic mapping of the presence of the white/no-white flowers in the genus Iris using All Rates Different model with 1,000 iterations. Pie charts represent the proportion of the iterations showing either presence or absence of the white/no-white flowers at any given node. [file Image_2.JPEG]

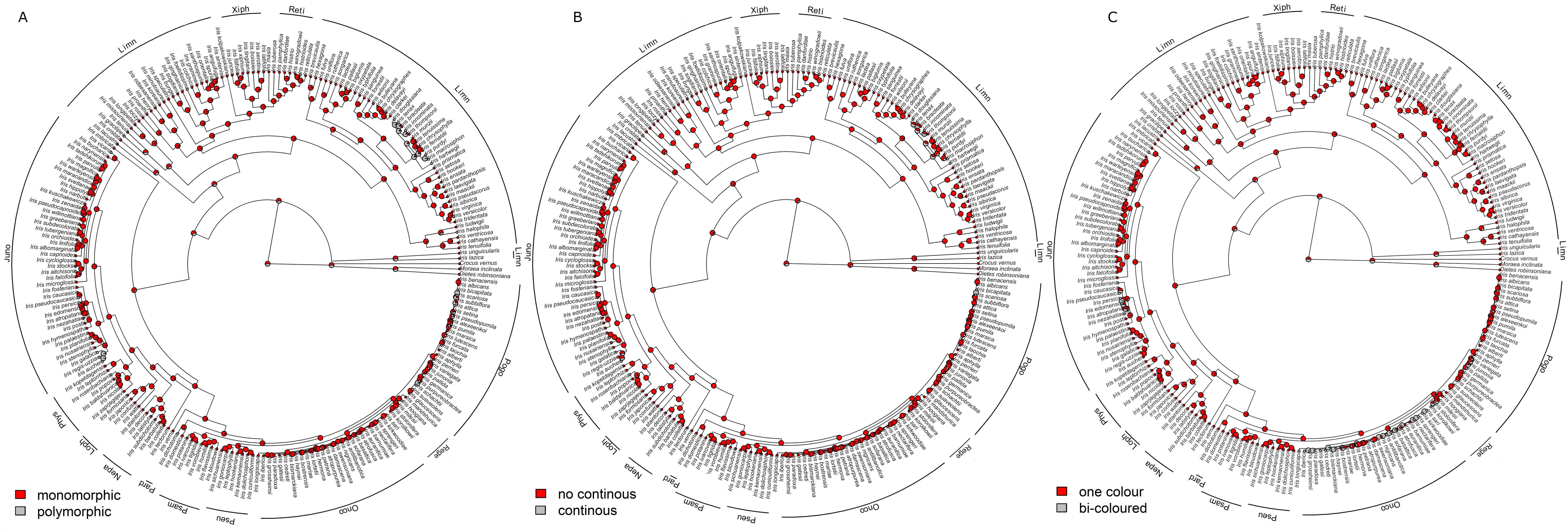

Supplement: Supplementary Figure 3 — Summarized stochastic mapping of the presence of the poly/monomorphic (A), continuous/non-continuous color (B), bi-colored/not bi-colored (C) flowers in the genus Iris using All Rates Different model with 1,000 iterations. Pie charts represent the proportion of the iterations showing either presence or absence of the studied traits in flowers at any given node. [file Image_3.JPEG]

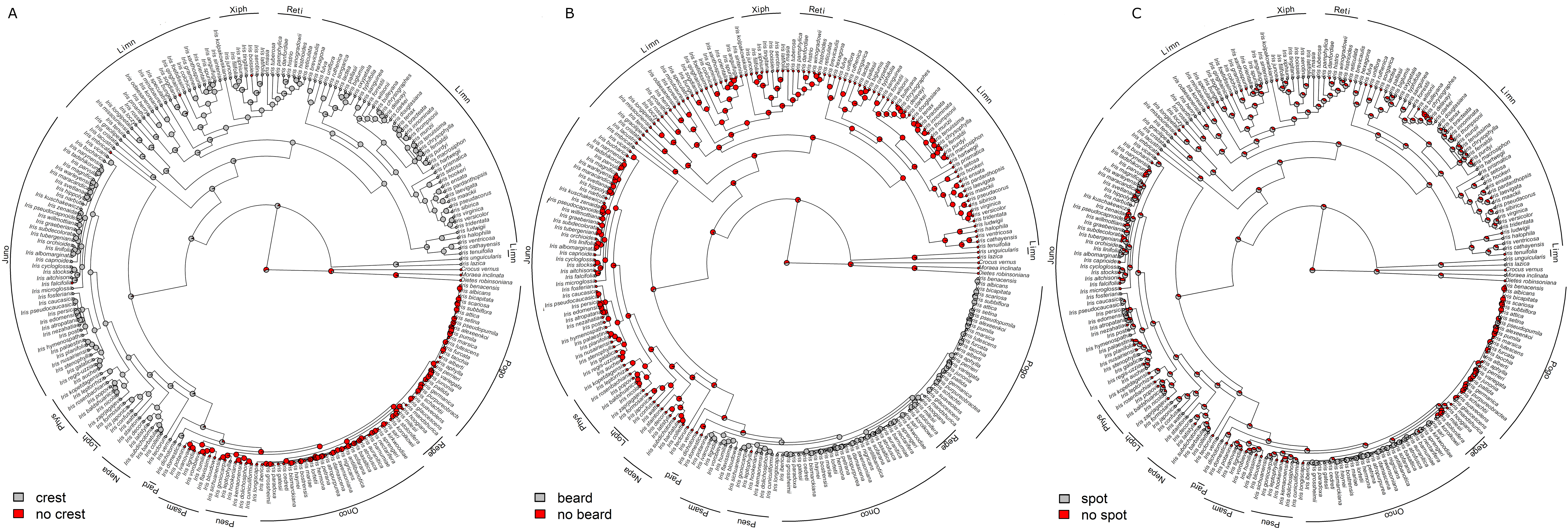

Supplement: Supplementary Figure 4 — Summarized stochastic mapping of the crest (A), beard (B), or spot (C) presence/absence in the genus Iris using All Rates Different model with 1,000 iterations. Pie charts represent the proportion of the iterations showing either presence or absence of studied trait at any given node. [file Image_4.JPEG]
