# Supplementary material for: All the Colors of the Rainbow: Diversification of Flower Color and Intraspecific Color Variation in the Genus Iris
Source: Front Plant Sci. 2020 Oct 13;11:569811. doi: 10.3389/fpls.2020.569811 (PMC7588356; doi:10.3389/fpls.2020.569811)
Supplement: Supplementary Material 2 — Evolutionary models selected with the use of ModelTest-NG program. [file Data_Sheet_2.PDF]

GTR+G4, IrisITS.aln.pre = 1-407
TVM+I+G4, IrismatK.aln.pre = 408-2016
GTR+I+G4, IrisndhF.aln.pre = 2017-4205
GTR+I+G4, IrisrbcL.aln.pre = 4206-4819
TVM+I+G4, IristrnK.aln.pre = 4820-5036
TPM1uf+I+G4, IristrnL.aln.pre = 5037-5899
