# Supplementary material for: All the Colors of the Rainbow: Diversification of Flower Color and Intraspecific Color Variation in the Genus Iris
Source: Front Plant Sci. 2020 Oct 13;11:569811. doi: 10.3389/fpls.2020.569811 (PMC7588356; doi:10.3389/fpls.2020.569811)
Supplement: Supplementary Material 3 — The contributing sub-trees of Iris genus; results and discussion of the phylogeny. [file Data_Sheet_3.pdf]

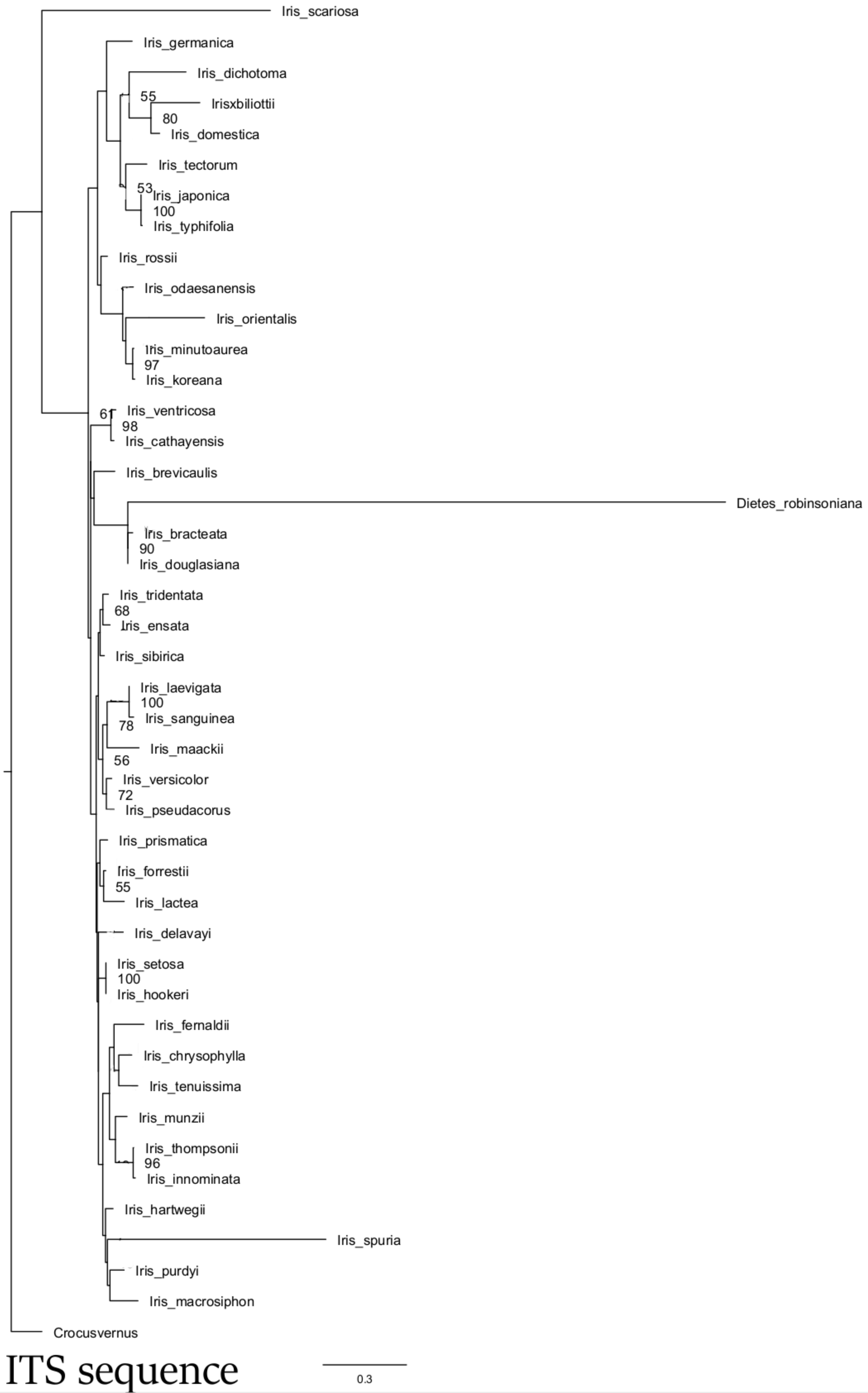

ITS sequence

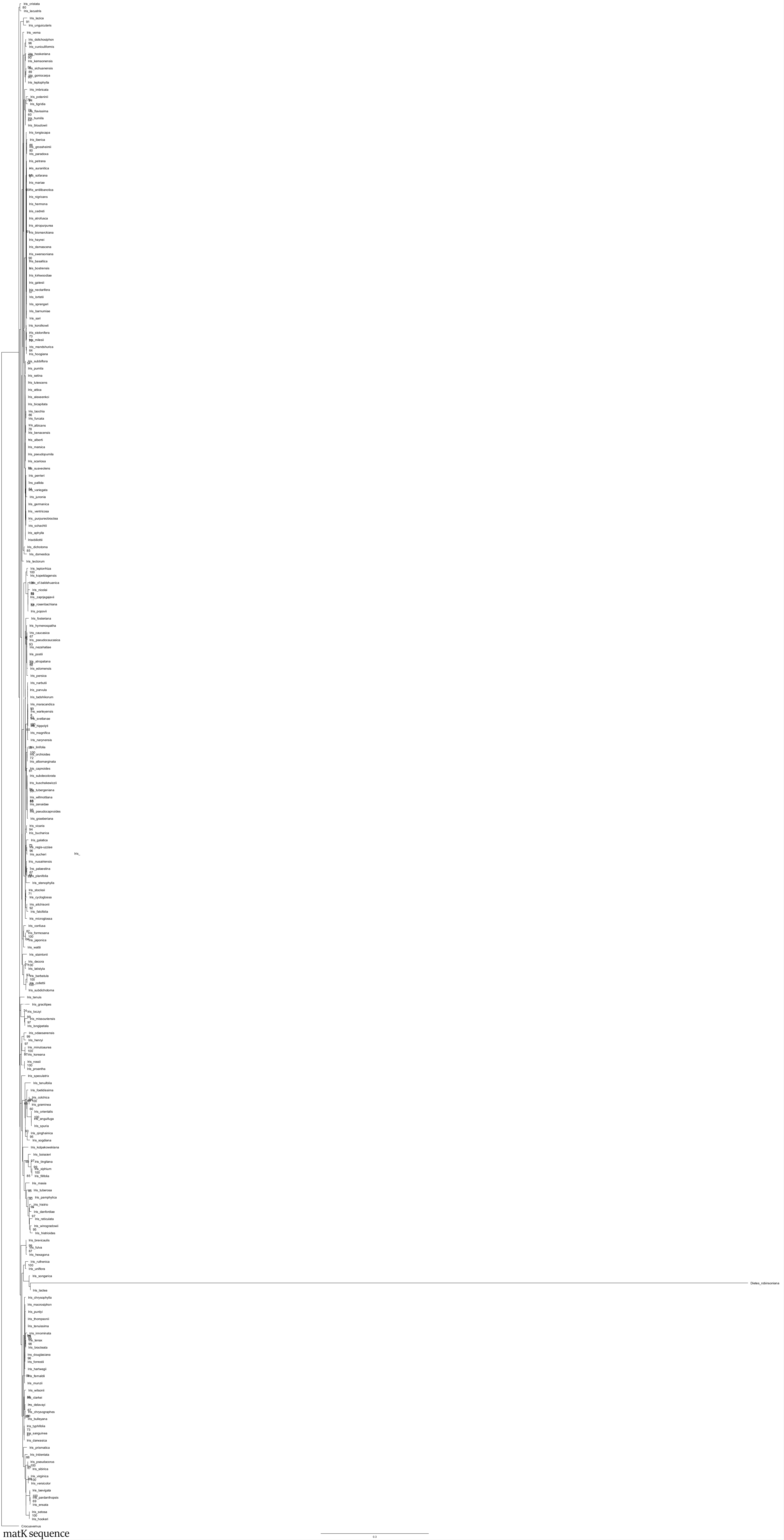

matK sequence

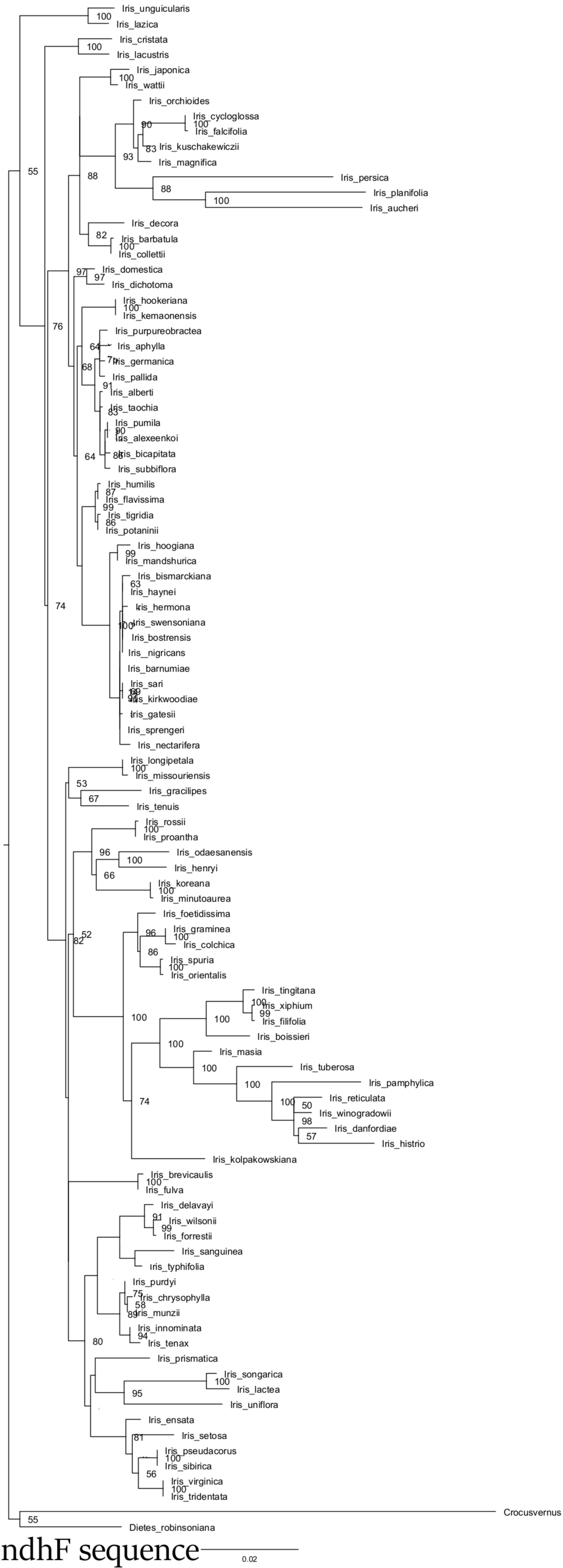

ndhF sequence

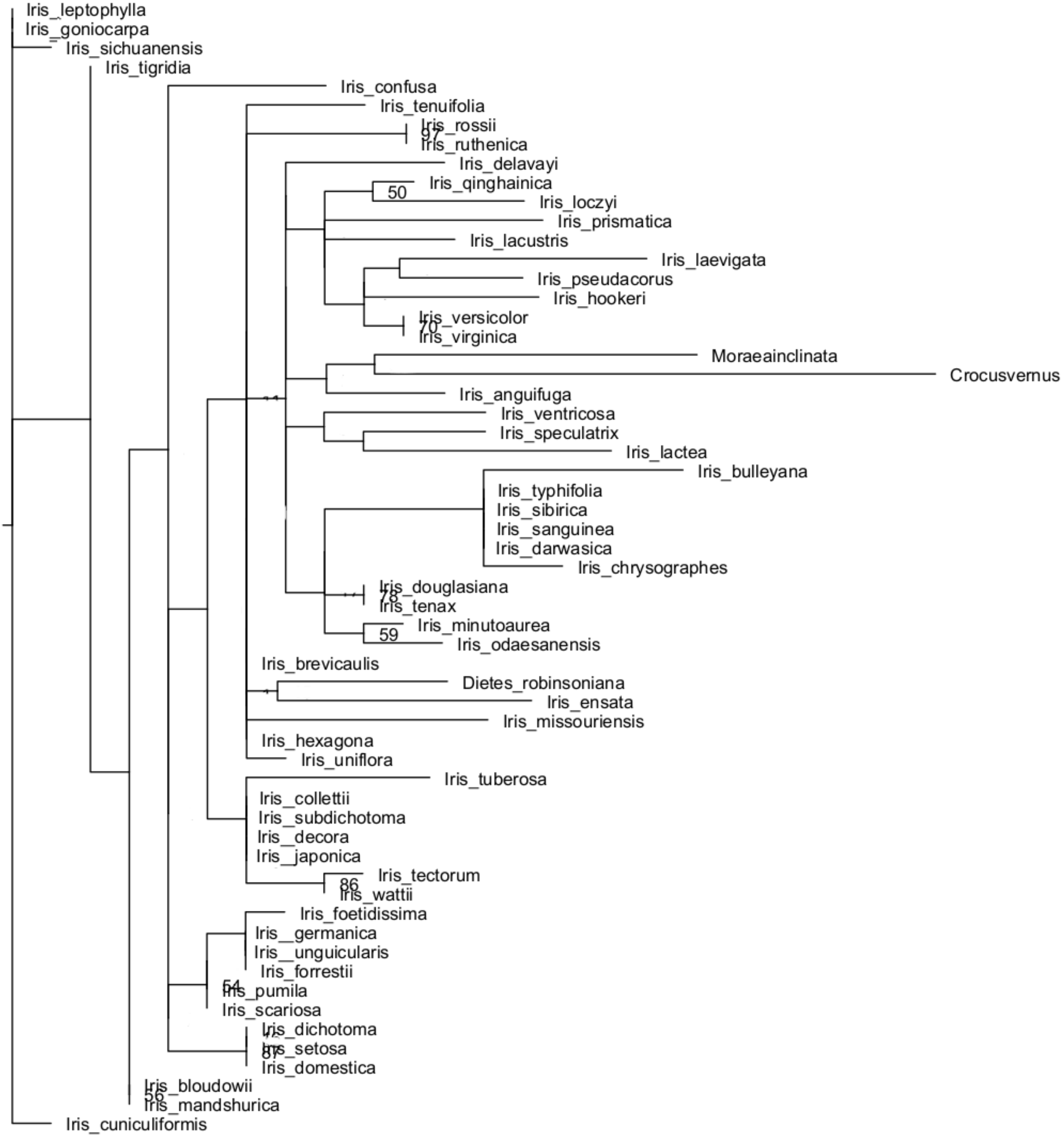

rbcl sequence

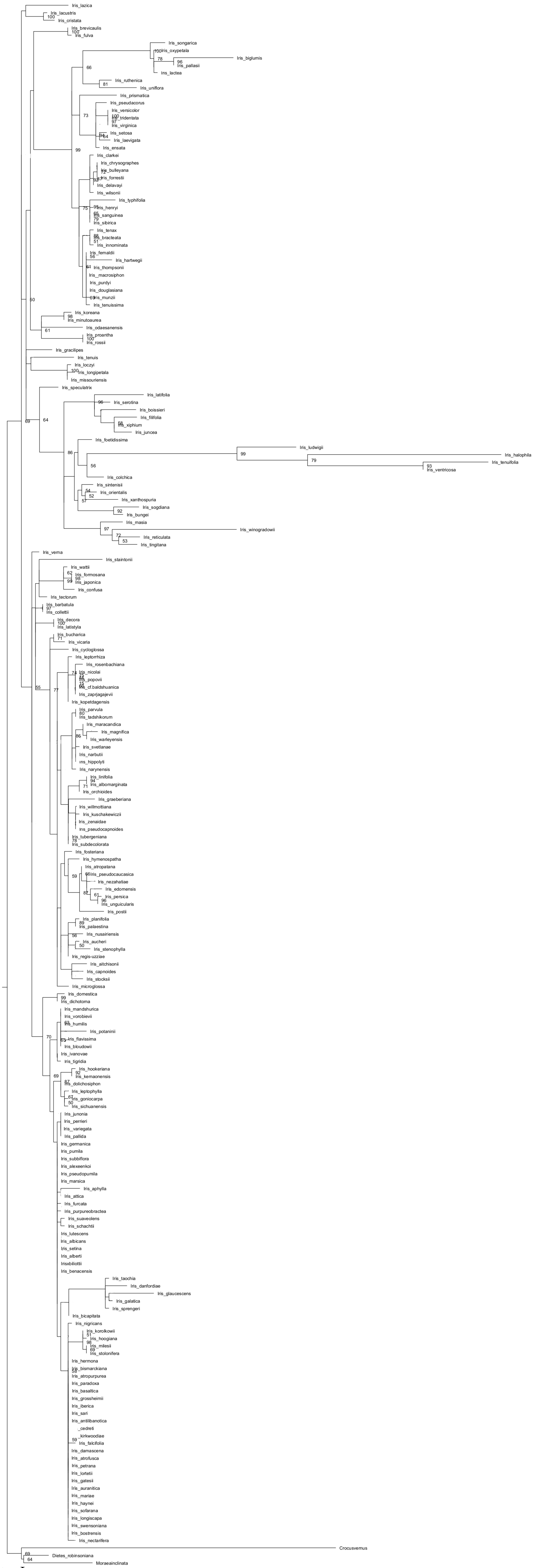

trnL seqence

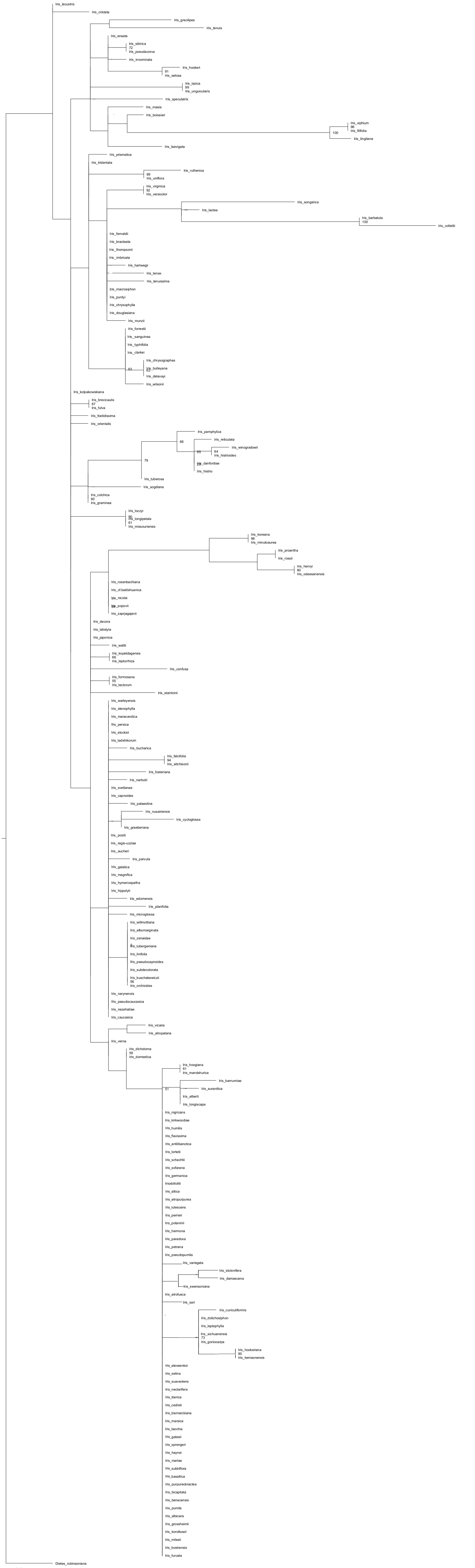

trnK sequence

Results

There were differences in the topology were observed between trees based on all six-locus trees and the trees based on each of used locus separately. In *mat*K and ITS trees, rooted on *Crocus vernus*, *Dietes robinsoniana* resolved as nested within subgenus *Limniris* (low bootstrap, hereafter Bp). Most of the described large-scaled relationships (Wilson, 2004; 2009; Wilson et al., 2016; Jiang et al., 2018) were recovered in present study.

The topology of all studied species evidenced two major subgenera (*Limniris* and *Iris*) and five minor (*Hermodactyloides, Nepalensis, Pardanthopsis, Scorpiris* and *Xiphium*). All but one genera were resolved as monophyletic, with *Hermodactyloides, Pardanthopsis* and *Scorpiris* being highly supported (Bp 86, 99 and 91 respectively). Only subgenus *Limniris* proved to be polyphyletic.

The *Scorpiris* subgenus (91 Bp), third by size and native to Middle East and Central Asia (Mathew, 1990), was represented by two highly supported sections *Juno* (80 Bp) and *Physocaulon* (100 Bp). This genus resolved as sister to two separated parts of subgenus *Limniris*.

Within subgenus *Iris,* distributed from eastern Asia through Southern or eastern Europe to North America (Mathew, 1990) six sections were resolved *Pardanthopsis* (99 Bp), *Psammiris* (56 Bp), *Pseudoregelia* (99 Bp), *Oncocyclus* (86 Bp), *Regelia* (87 Bp), *Hexapogon* (86 Bp) and *Pogon*. *Iris falcifolia*, described as belonging to the section *Hexapogon* was resolved in the section *Limniris*. Also *I. tigridia* and *I. ivanovae* classified as representing section *Pseudoregelia* resolved in section *Psammiris*. *Iris milesii* described as part of the subgenus *Limniris* resolved in the section *Pseudoregelia*. *Iris mandsuhgarica* described in *Psammiris* resolved in *Pogon*.

Within *Limniris,* genera widely distributed around Northern Hemisphere (Mathew, 1990), two sections were resolved *Limniris* (71 Bp) and *LophIris*, with the first section containing the majority of the species belonging to genera *Limniris*. Several species classified as belonging to section *LophIris* resolved as part of the section *Limniris.*

The subgenera *Hermodactyloides* (86 Bp), distributed from East Europe to Central Asia (Mathew, 1990), is subdivided into two sections *Monolepsis* (represented by one species, 73 BP*),* and *Reticulatae* (86 Bp). A sister subgenus *Xiphium* (97 Bp), distributed in western Europe and North Africa, contains only *Xiphium* section.

The subgenus *Nepalensis,* (58 Bt), distributed only in south-western China (Mathew, 1990) and represented by *Nepalensis* section, resolved as closely related to *Pardanthopsis* (99 Bp),

the subgenus native to Asia (Mathew, 1990) and represented by two species. *Iris subdichotoma* described as species in section Loph*Iris* resolved in the section *Nepalensis. Iris lazica* and *I. unguicularis* resolved as separated subgenus, with high support (100 Bp) similarly as *I. imbricata* (78 Bp).

Discussion

The phylogeny presented in this paper is the most comprehensive study of genus *Iris*. The topology of obtained trees is in most cases consistent with previously published data (Tillie et al., 2000; Makarevitch et al., 2003; Wilson, 2009; Wilson, 2011; Jiang et al., 2018). In some cases, using *trn*L-F may have resulted in lack of bootstrap support of some branches, which was described by Tillie et al. (2000). Previously described generic groupings are recovered in present study as subgenera and subsections (Tillie et al., 2000; Makarevitch et al., 2003; Wilson, 2009; Wilson, 2011; Jiang et al., 2018), with monophyletic subgenera *Hermodactyloides, Iris, Nepalensis, Pardanthopsis, Scorpiris* and *Xiphium*, and paraphyletic *Limniris*. This study, similar like Wilson (2011), resolved *I. dichotoma* and *I. domestica* as sister taxa in subgenus *Pardanthopsis* that is sister to subgenus *Iris*. Similarly like in Wilson (2011) study *I. falcifolia* resolved as species belonging to subgenus *Scorpiris.* Contrary to previous works (Dykes, 1913; Mathew, 1989; Wilson, 2011) *I. tuberosa* was recognized as species belonging to subgenus *Limniris*.

References

Dykes, W.R. (1913). *The Genus Iris.* New York: Dover Publications.

Jiang, Y.L., Huang, Z., Liao, J.Q., Song, H.X., Luo, X.M., Gao, S.P., et al. (2018). Phylogenetic analysis of IRIS L. from China on chloroplast TRNL-F sequences. *Biologia (Poland)* 73(5)**,** 459-466. doi: 10.2478/s11756-018-0063-0.

Makarevitch, I., Golovnina, K., Scherbik, S., and Blinov, A. (2003). Phylogenetic relationships of the siberian *Iris* species inferred from noncoding chloroplast DNA sequences. *International Journal of Plant Sciences* 164(2)**,** 229-237.

Mathew, B. (1990). *The iris.* Portland, Ore.: Timber Press.

Tillie, N., Chase, M.W., and Hall, T. (2000). Molecular studies in the genus Iris L.: A preliminary study. *Annali di Botanica* 58(March)**,** 105-112. doi: 10.4462/annbotrm-9068.

Wilson, C. (2006). Patterns in Evolution in Characters That Define Iris Subgenera and Sections. *Aliso* 22(1)**,** 425-433. doi: 10.5642/aliso.20062201.34.

Wilson, C.A. (2004). Phylogeny of *Iris* based on chloroplast matK gene and trnK intron sequence data. *Molecular Phylogenetics and Evolution* 33(2)**,** 402-412. doi: http://dx.doi.org/10.1016/j.ympev.2004.06.013.

Wilson, C.A. (2009). Phylogenetic relationships among the recognized series in *Iris* section Limniris. *Systematic Botany* 34(2)**,** 277-284.

Wilson, C.A. (2011). Subgeneric classification in Iris re-examined using chloroplast sequence data. *Taxon* 60(1)**,** 27-35.

Wilson, C.A., Padiernos, J., and Sapir, Y. (2016). The royal irises (Iris subg. Iris sect. Oncocyclus): Plastid and low-copy nuclear data contribute to an understanding of their phylogenetic relationships. *Taxon* 65(1)**,** 35-46. doi: 10.12705/651.3.

Winkel-Shirley, B. (2002). Biosynthesis of flavonoids and effects of stress. *Current Opinion in Plant Biology* 5(3)**,** 218-223. doi: http://dx.doi.org/10.1016/S1369-5266(02)00256-X.
